# Current Strategies to Modulate Regulatory T Cell Activity in Allergic Inflammation

**DOI:** 10.3389/fimmu.2022.912529

**Published:** 2022-05-26

**Authors:** Iris Bellinghausen, Rahul Khatri, Joachim Saloga

**Affiliations:** Department of Dermatology, University Medical Center, Johannes Gutenberg-University Mainz, Mainz, Germany

**Keywords:** allergic inflammation, regulatory T cells, allergen-specific immunotherapy, microbiota, Treg engineering, therapeutic application

## Abstract

Over the past decades, atopic diseases, including allergic rhinitis, asthma, atopic dermatitis, and food allergy, increased strongly worldwide, reaching up to 50% in industrialized countries. These diseases are characterized by a dominating type 2 immune response and reduced numbers of allergen-specific regulatory T (Treg) cells. Conventional allergen-specific immunotherapy is able to tip the balance towards immunoregulation. However, in mouse models of allergy adaptive transfer of Treg cells did not always lead to convincing beneficial results, partially because of limited stability of their regulatory phenotype activity. Besides genetic predisposition, it has become evident that environmental factors like a westernized lifestyle linked to modern sanitized living, the early use of antibiotics, and the consumption of unhealthy foods leads to epithelial barrier defects and dysbiotic microbiota, thereby preventing immune tolerance and favoring the development of allergic diseases. Epigenetic modification of Treg cells has been described as one important mechanism in this context. In this review, we summarize how environmental factors affect the number and function of Treg cells in allergic inflammation and how this knowledge can be exploited in future allergy prevention strategies as well as novel therapeutic approaches.

## Introduction

The prevalence of allergic and of most autoimmune diseases like diabetes, inflammatory bowel disease, multiple sclerosis, and rheumatoid arthritis, has dramatically increased worldwide, reaching epidemic dimensions especially in industrialized countries. The immunological mechanisms of (atopic) allergic diseases, that is, allergic rhinitis, asthma, atopic dermatitis, insect venom allergy, and IgE-mediated food allergy, are characterized by an aberrant type 2 immune response to otherwise harmless allergens and a decrease in allergen-specific regulatory T (Treg) cells that are restored during allergen-specific immunotherapy (AIT), the only causal-oriented treatment known so far. However, AIT is cumbersome and not established for all kinds of allergies. Therefore, the clinical demand for other therapies remains high. In this review article, we discuss novel treatment strategies to restore the function of Treg cells in allergic inflammation by either boosting Treg cell expansion *in vivo* or by reinfusion of isolated and *ex vivo* engineered Treg cells.

## Mechanisms of Allergic Inflammation

During sensitization against a specific allergen, several cell types of the innate and adaptive immune system and various ligand-receptor-interactions are triggered (1–3). Initially, the allergens traverse epithelial barriers and are internalized by antigen-presenting cells (APC), especially DC, located in the skin, airways and gut mucosa. According to the epithelial barrier hypothesis, an increase in epithelial barrier-damaging agents due to industrialization like toxins, surfactants and emulsifiers in household cleaning agents or processed food, cigarette smoke, particulate matter, diesel exhaust particles, ozone, nanoparticles and microplastics, is associated with the rise in allergic, autoimmune and other chronic inflammatory diseases. Protease activity of allergens as well as exposure to certain bacteria and viruses was also shown to facilitate disruption of tight junction proteins further leading to epithelial barrier leakiness (4). Damaged epithelial cells release alarmins (IL-25, IL-33, thymic stromal lymphopoietin (TSLP)), which activate DC and group 2 innate lymphoid cells (ILC2). Activated DC migrate to local lymph nodes where they present processed allergen peptides to naive CD4^+^ T cells on MHC class II molecules. Recognition of these peptide/MHC complexes by the T-cell receptor induces differentiation of T helper cells with a Th2-type cytokine profile (IL-4, IL-5, IL-9, and IL 13) depending on additional costimulatory signals from the DC and the surrounding micromilieu. Furthermore, IL-5, IL-13, and IL-9 are produced by ILC2 (4). Expression of IL-4 and IL-13 together with ligation of suitable costimulatory molecules (CD40 with CD40 ligand, and CD80 or CD86 with CD28) induces an immunoglobulin class switch and the production and secretion of IgE antibodies by B cells. IgE is bound to the surface of effector cells such as mast cells and basophils *via* the high affinity IgE-receptor (FcϵRI). In sensitized individuals, crosslinking of these IgE-FcϵRI-complexes after re-exposure to the same allergen rapidly leads to release of pro-inflammatory mediators like histamine, tryptase, prostaglandins, leukotrienes, and cytokines, which cause the first early phase response with typical allergic symptoms such as rhinorrhea, airway mucus secretion, broncho-constriction as well as urticaria, vomiting, and diarrhea in the case of food allergies and even leading to anaphylaxis. The late phase response, characterized by the recruitment of further mast cells and eosinophils mainly induced through IL-5, follows within several hours. During the repeated exposure the allergen is also taken up by DC, thereby activating memory Th2 cells to produce cytokines and other mediators, which may further lead to chronic airway inflammation, goblet cell hyperplasia and tissue remodeling (3, 5, 6).

## Treg Cell Subsets Involved in Allergic Inflammation and Ait

The concept of immune tolerance by specialized lymphocytes to self-antigens has long been suspected but only proven after the identification by *Sakaguchi* et al. that a small population of CD25-expressing CD4^+^ T cells could prevent autoimmunity in mice (7). A second breakthrough was the discovery of the transcription factor FoxP3 as master regulator of Treg cell differentiation and function. Mutations or deletion of this gene cause severe autoimmune disease known as *scurfy* phenotype in mice and IPEX (immune dysregulation, polyendocrinopathy, enteropathy, X-linked) syndrome in humans which suffer from diabetes mellitus, high serum IgE levels, eosinophilia, and food allergy (8–10). CD4^+^CD25^high^CD127^low^ Treg cells constitute about 5-7% of circulating CD4^+^ T cells, and they are either generated in the thymus (tTreg) or induced in the periphery (pTreg), especially in the gut, from conventional T cells in the presence of TGF-ß or retinoic acid (11–13). While tTreg constitutively express the transcription factor FoxP3, which is essential for their differentiation and function, pTreg can lose FoxP3 expression and convert into IL-17- and IFN-γ-producing inflammatory effector T cells (14). In general, Treg cells possess remarkable plasticity in response to a changing cytokine milieu by expressing alternate lineage transcription factors such as T-bet, GATA3 and RORCγt (15, 16). Furthermore, at least in humans, FoxP3 is also transiently expressed by activated effector T cells. Stability of FoxP3 expression relies on DNA hypomethylation of the Treg cell-specific demethylated region (TSDR) within the conserved non-coding sequence 2 (CNS2) in the Foxp3 gene region (17). Helios and neuropilin-1 are also almost exclusively expressed in tTreg compared to pTreg (18, 19). Another population of Treg cells, namely CD49b^+^LAG-3^+^ T regulatory type 1 (Tr1) cells, can be induced in the periphery upon antigen exposure under tolerogenic conditions which exert their suppressive capacity independently from FoxP3 mainly *via* secretion of IL-10 (20–22).

Treg cells play a critical role in maintaining immune tolerance to allergens and all above-mentioned Treg cell subsets have been described to control type 2 immune cells by producing inhibitory cytokines such as IL-10, TGF-ß and IL-35 (**Table 1**). Furthermore, Treg cells express high-levels of surface markers associated with suppression like CTLA-4, PD-1, GITR, ICOS, LAP, GARP, TIM-3, TIGIT, CD39 and CD73, and suppress effector T cells through cell-cell-contact, cytolysis and metabolic disruption, reviewed in (31). Although we and others have shown that the suppressive capacity of peripheral blood derived Treg cells from allergic individuals is not generally defective compared to healthy controls, it may be reduced under certain conditions (32–34). For example, Treg from grass-pollen allergic donors failed to inhibit proliferation of T effector cells at high allergen doses while Treg cells from non-allergic donors did not fail at these allergen concentrations, probably due to production of high amounts of IL-2 by responder T cells following increased stimulation thereby escaping suppression (35, 36). The fact that only a few soluble proteins from inhaled allergens are Th2 and IgE inducers/targets, while the vast majority of encountered proteins attached to inhaled particles is still tolerated, also argues against a global defect in Treg cell deficiency in allergic patients (37). Nevertheless, on the other hand, recent reports have shown that tissue derived Treg cells are quantitatively reduced and functionally impaired in asthmatic children (38). Furthermore, patients with allergic rhinitis have lower percentages of antigen-specific Tr1 cells in peripheral blood compared to healthy donors, and induced peanut-specific Tr1 cells from allergic patients are functionally defective (39, 40). Importantly, improvement of allergic symptoms in successful AIT, characterized by repeated subcutaneous or sublingual applications of increasing doses of the specific allergen, is associated with an increased number of allergen-specific Treg cells (**Table 1**) as well as and Breg cells (23, 41, 42). In contrast, the recently defined dysregulated FoxP3^+^ILT3^+^ Treg cell subset, occurring in a higher frequency in asthmatic compared to healthy donors, is reduced during AIT (**Table 1**) (30, 43). Increased antigen-induced regulatory T-cell function has also been observed after peanut or milk oral immunotherapy (OIT) (25, 44). Overall, a decrease in allergen-specific IgE together with an increase in protective IgG4 antibodies is achieved (23, 26, 41, 42). Furthermore, Treg have been demonstrated to directly block mast cell degranulation and ILC2 through OX40-OX40L or ICOS-ICOSL interaction, respectively (45, 46).

**Table 1:** Treg cell subsets involved in allergic inflammation and increased (green) or decreased (red) during AIT or OIT.

	Specific markers	Cytokines	References
**tTreg cells**	CD4, CD25, CD127^low^, FoxP3, Helios, neuropilin-1, CTLA-4, ICOS, GITR, PD-1, CD39, CD73, GARP, LAP	IL-10, TGF-ß, IL-35	(23, 24)
**pTreg cells**	CD4, CD25, CD127^low^, FoxP3, CTLA-4, ICOS, GITR, PD-1, CD39, CD73	IL-10, TGF-ß, IL-35	(25–27)
**Tr1 cells**	CD4, CD25, LAG-3, CD49b, PD-1, CTLA-4, ICOS, TIGIT, TIM-3	IL-10, TGF-ß	(20, 28)
**RORγt^+^ Treg cells in the gut**	CD4, CD25, FoxP3, RORγt, CTLA-4, ICOS, GITR, CD39, CD73, CCR6	IL-10, IL-17	(29)
**ILT3^+^ Treg cells**	CD4, CD25, FoxP3, ILT3	IL-5, IL-13	(30)

## Impact of Microbial Metabolites and Diet on Treg Function in Allergic Inflammation

Despite a genetic predisposition or other risk factors like recurrent upper respiratory tract infections in early life, it is becoming increasingly clear that the increased incidence of allergic diseases is related to changes in the mucosal microbiome and to mucosal barrier defects. These changes are due to modern sanitized living with reduced exposure to environmental microorganisms, as suggested by the so-called hygiene hypothesis (47, 48), as well as to the consumption of unhealthy foods, high in fat, sugar and certain food additives, and low in fiber (49). The importance of the microbiota for immune tolerance has been demonstrated in mice raised under germ-free conditions which display strongly reduced Treg cell frequency in the gut and are more susceptible to develop allergies (50, 51). Recolonization of the gut with commensal bacteria such as *Firmicutes*, *Bacteroidetes*, *Bifidobacterium* and *Prevotella* restored Treg cell numbers *via* different mechanisms (52). For instance, fiber is fermented to short chain fatty acids (SCFA), and it has been demonstrated in several animal models and in human studies that the most abundant SCFA acetate, propionate and especially butyrate can promote Treg cell differentiation and function, thereby preventing or ameliorating the development of food and inhalational allergy (53–55). Butyrate has also been shown to ameliorate ILC2-driven airway inflammation (56). These effects appeared to be mediated by SCFA-induced G-protein-coupled receptors (GPCR) activation, mainly GPR41, GPR43 and GPR109A, by activation of transcription factors such as aryl hydrocarbon receptor (AhR), or *via* epigenetic modification through inhibition of histone deacetylases (HDAC). Vitamin A and its metabolite retinoid acid further induce Treg cell development and a combination of both vitamin A and dietary fiber was required to maintain protection against food allergy (53, 57–60). In addition, consumption of unsaturated omega-3 fatty acids present for example in olive oil and fish has also been associated with reduced risk of allergic rhinitis and asthma (61). Molecules like polysaccharide A (PSA) produced by *Bacteroides fragilis* can also boost Treg cell generation *via* Toll-like receptor 2 (TLR2) signaling (62). Furthermore, tryptophan metabolites derived from intestinal commensals or catabolized by indoleamine 2,3-dioxygenase (IDO)-expressing tolerogenic DC ameliorate allergic responses (63). In contrast, wheat amylase trypsin inhibitors (ATI), activators of intestinal myeloid cells *via* TLR4 and promoters of microbial dysbiosis (64, 65), have been shown to enhance allergic intestinal and airway inflammation (66, 67). Importantly, it has recently been reported that high frequencies of RORCγt^+^ Treg are required to tolerate the intestinal microbiota and to prevent inflammatory type 2 diseases in the gut (68). Furthermore, RORCγt^+^ Treg possess a protective role in a model of food allergy (69). Altogether, a disturbed microbiota favors intolerances to food-derived antigens, whereas administration of probiotics may promote protection from allergic intestinal and airway inflammation (49, 59, 70).

## Current Therapeutic Applications in Allergic Diseases: I, Induction or Expansion of Treg *in Vivo*

**Figure 1:**
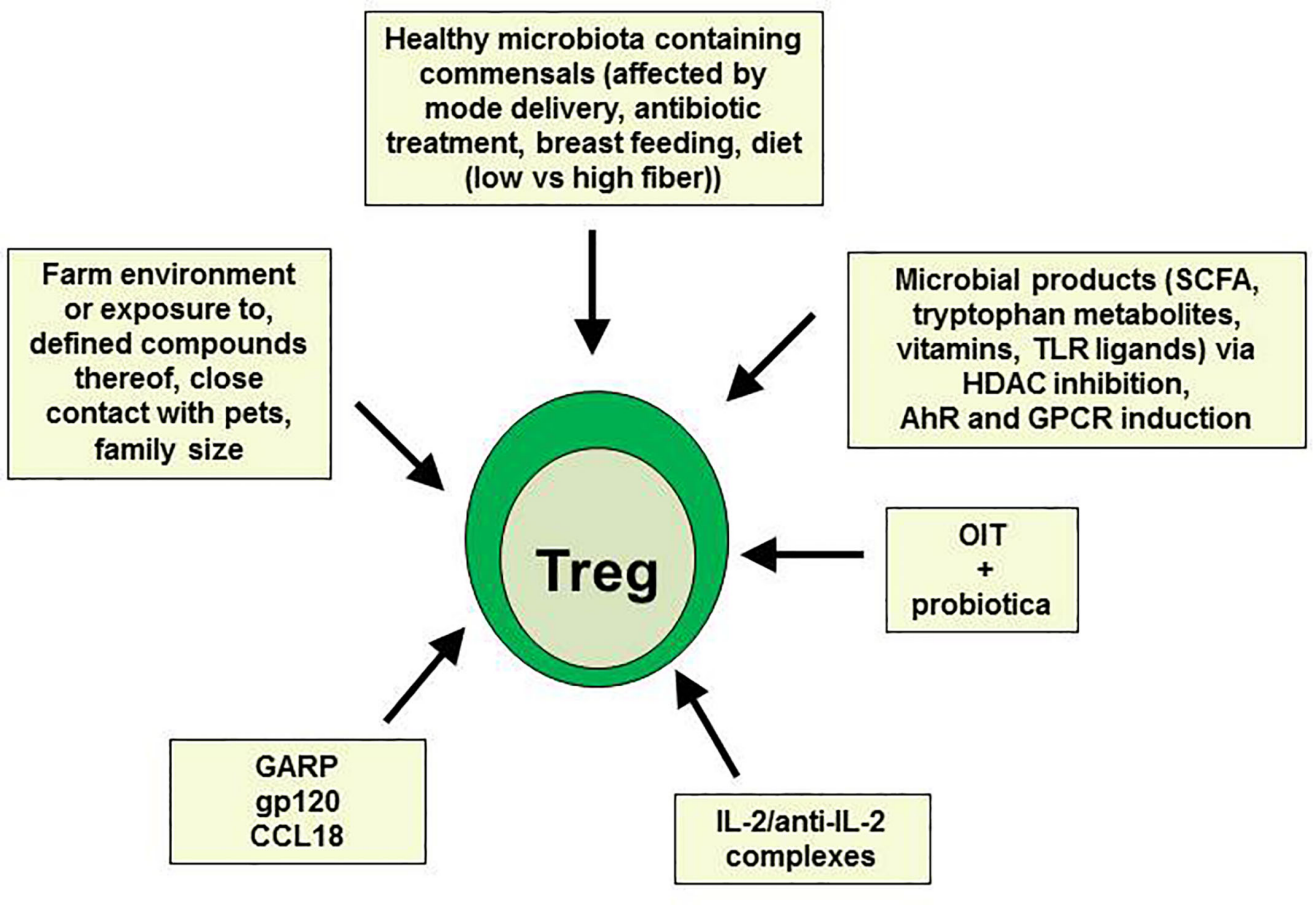
Prevention of type 2 allergic responses by *in vivo* expansion of Treg.

As already mentioned above, allergen-specific Treg cells are induced during successful AIT. However, AIT is not yet applicable for all kinds of allergies, and did not lead to beneficial results in all patients. Therefore, other therapeutic settings aiming to induce or expand Treg cells are urgently needed (summarized in **Figure 1**). Using a humanized mouse model of allergy, where PBMC from highly sensitized allergic donors were injected into immunodeficient nonobese diabetic-severe combined immunodeficiency gamma chain knockout (NSG) mice, we have recently shown that Treg cells can be induced by administration of the chemokine CCL18 produced by tolerogenic IL-10 DC (71–73) or soluble GARP (74, 75). Both factors led to inhibition of allergen-specific human IgE production in mouse sera and subsequently to prevention of allergen-driven IgE-dependent airway and intestinal inflammation (73–75). GARP has formerly been described to be up-regulated on activated Treg and to mediate their suppressive function (76, 77). In addition, administration of soluble GARP prevented the onset of a xenogeneic graft-versus-host disease (GvHD) in NSG mice being injected with human PBMC directly after birth also by enhancing Treg cell activity (78). In both models, the suppressive function of Treg could further be enhanced by the CD4-binding HIV-1 surface protein gp120, preventing GvHD or allergic airway inflammation, respectively (79, 80). Recently, it has been reported that low-dose IL-2 or a combination of IL-2/anti-IL-2 restored Treg numbers and function in respiratory and food allergies, thereby improving allergic markers and symptoms (81–83).

Manipulation of the intestinal microbiota associated with induction or expansion of Treg cells as shown in numerous animal models of allergy and asthma may also offer novel strategies for immunomodulation of food allergies. However, only a slight improvement could be observed in clinical studies so far. For example, in a double-blind, placebo-controlled clinical trial, co-administration of the probiotic *Lactobacillus rhamnosus* reduced peanut-specific IgE levels during peanut oral immunotherapy while peanut-specific IgG4 levels were increased (29). Additionally, colonization of mice and humans with certain Clostridia species has been shown to protect from food allergy *via* increasing Treg cell numbers (60, 69). In a more recent study, fecal transplantation from healthy but not cow’s milk allergic infants successfully prevented anaphylactic responses in susceptible germ-free mice, thus confirming the importance of intestinal bacteria in promoting tolerance to dietary antigens (84). Importantly, the early-life microbiota established in uterus and during the first months of life plays a crucial role for proper immunological development and overall health status (85). In this respect, an increased risk of the development of food allergy and asthma has been reported for children born by caesarian instead of vaginal delivery. This may be due to a direct impact of the vaginal microbiota being rich in *Lactobacilli* species on the microbiota development of the baby (86, 87). Perinatal antibiotic prophylaxis after caesarian delivery or the absence of stress factors associated with a vaginal delivery may also contribute to this effect. In general, a strong relationship between increased antibiotic use in the first few years of life and allergy development has been reported (88). Antibiotics not only reduce important protolerogenic bacteria, they also induce metabolic changes and enhance susceptibility to obesity (60, 89). Furthermore, *Lactobacilli* and *Bifidobacteria* as well as oligosaccharides in the breast milk are important triggers of a balanced healthy immune system, and longer duration of breastfeeding is associated with Treg cell expansion and reduced allergy development (90–92). In addition, a dietary diversity in the first years of life and early consumption of peanuts or eggs as well as other factors relating to the hygiene hypothesis, like living in a farm environment and drinking farm milk, or exposure to a large number of pets, both associated with an enhanced microbial diversity, have been described to protect against allergy and asthma, particularly *via* the induction of Treg cells (93–99). For example, chronic exposure to low-dose endotoxin or farm dust has been shown to inhibit developing house dust mite-induced asthma in mice (100). Of note, the allergy protective farm effect was previously imitated by the lipocalin beta-lactoglobulin (BLG) present in cow’s raw milk and farm dust when loaded with iron-flavonoid complexes. Holo-BLG promoted Treg cells through AhR activation and down-regulation of antigen presentation in monocytes and DC *via* transport of iron (101). In a follow-up clinical pilot trial, the same authors assessed the efficacy of a FSMP (food for special medical purposes) lozenge containing BLG with iron, polyphenols, retinoic acid, and zinc in allergic women and could show that this holo-BLG lozenge improved nasal symptom score after nasal provocation as well as symptom medication score during birch or grass pollen season by more than 40% compared to the placebo group (102).

However, more and larger studies are needed to confirm all these positive results and to develop public health strategies to prevent allergic diseases in the future.

## Current Therapeutic Applications in Allergic Diseases: II, Infusion of *Ex Vivo* Engineered Treg

The therapeutic potential of FoxP3^+^ Treg cells has been demonstrated in many preclinical models and clinical trials aiming to prevent graft-versus-host disease during organ transplantation and in several autoimmune diseases such as type 1 diabetes mellitus, inflammatory bowel disease, systemic lupus erythematosus, multiple sclerosis, and allergy (103). Tr1 cells therapy in the context of allogeneic hematopoietic stem cell transplantation has also been successfully employed (104). Overall, the functional stability of Treg cells is of fundamental importance for the efficacy and safety of Treg cell-based therapies.

Gene editing technologies in medical research are becoming a central player to investigate and treat complex biological diseases. Constant balanced examination of the immune system of autonomous and non-autonomous antigens protects healthy cells and eliminates invading foreign particles. However, failing in recognition of self and non-self antigens leads to various autoimmune and inflammatory diseases such as inflammatory bowel disease, type 1 diabetes, rheumatoid arthritis and multiple sclerosis (105). Standard medication for various autoimmune diseases including allergy required immunosuppression in a form of pharmacological compounds and antibody targeted treatment which subjected patients to other complications such as cancer and opportunistic pathogens. In this regard, cell-based therapy such as adoptive cell transfer of engineered Treg cells could provide an effective therapeutic alternative to combat autoimmune diseases.

**Figure 2:**
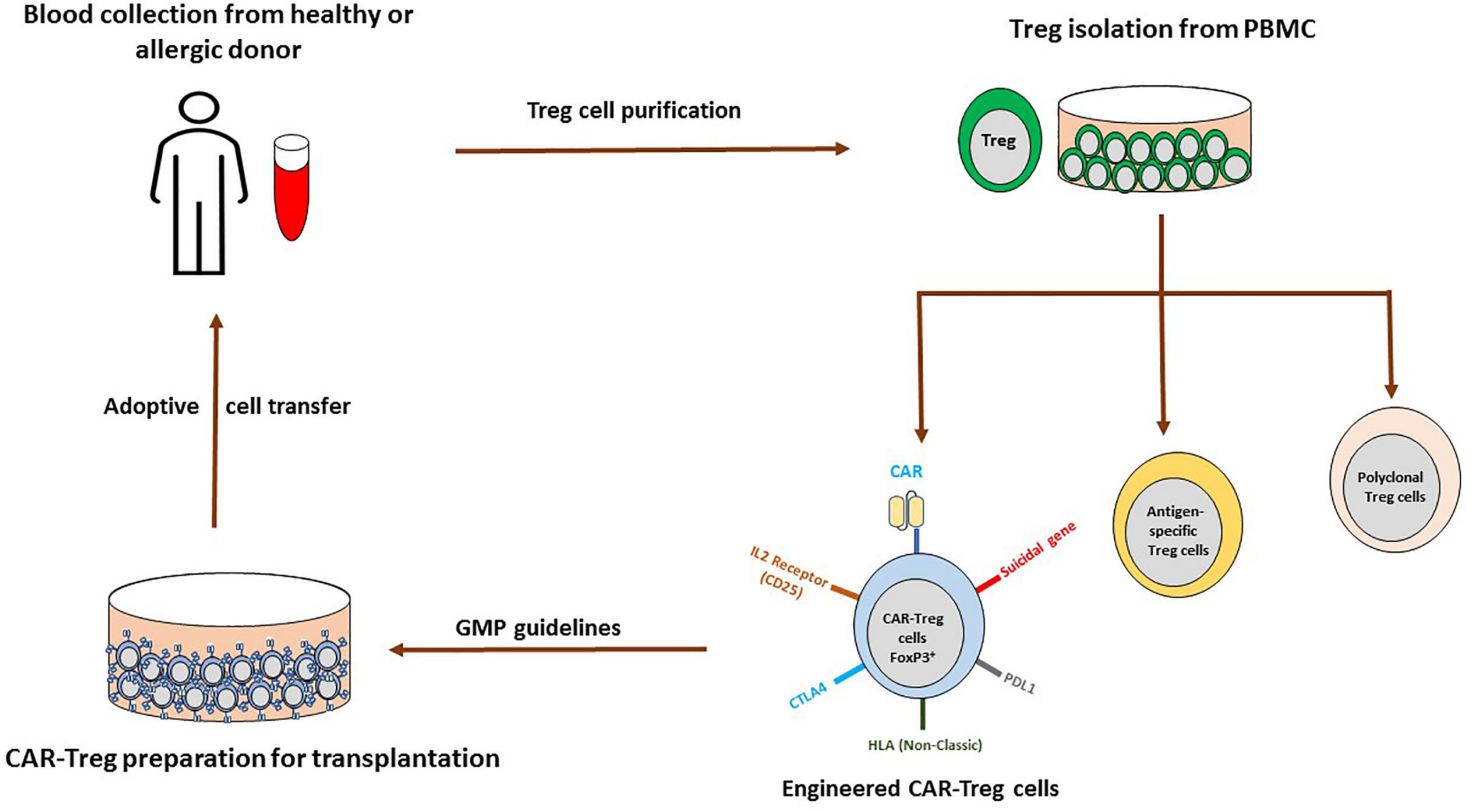
Possible adoptive CAR-Treg cell therapy for allergic asthma. Treg cells are isolated from peripheral blood of healthy and allergic donor, cultured with IL-2 for polyclonal Treg cells (CD3/CD28 beads), antigen-specific cells (antigen presenting cells activate alloreactive Treg cells) and genetically engineered Treg cells. In genetically engineered Treg approach, cells could be transduced with retroviruses, lentiviruses, adenovirus-associated virus (AAV), transcription activator-like effector nucleases (TALEN), Zinc finger nucleases (ZFN) or CRISPR-Cas to express CAR, TCR, BAR and others. With this approach immunological rejection would be avoided (introduction of the non-classical HLA and deletion of donor HLA molecules), in case of off target, suicidal gene system could be activated and co-expression of others such as PDL1, CTLA4, and IL-2R would help in the specific migration, suppression and inactivation of T cells. Further, following GMP regulations, expanded cells could be infused in the patients.

Adoptive cell transfer of Treg cells is effective and well tolerated. For instance, in a phase I/IIa clinical trial investigating Treg cell therapy in kidney transplantation, patients engrafted with autologous Treg had similar rejection rates compared to the group receiving the standard immunosuppression with basiliximab (anti-CD25), but displayed reduced infection rates (106). In clinical settings, polyclonal expansion, antigen-specific stimulation and engineered (CAR: chimeric antigen receptor and TCR: predefined T cell receptor) Treg cells are the prime strategies. In polyclonal expansion, Treg cells from donor PBMC were activated in the presence of anti-CD3/28 beads and IL-2 whereas an antigen-specific approach employed allogeneic Treg cells from the recipient stimulated by antigen presenting cells from the graft donor. However, an antigen-specific approach is more efficient with higher specificity than polyclonal Treg cells and suitable for the transplantation. Engineered approaches use genetic modification of polyclonal Treg cells with CAR or TCR to recognize desired antigens and provides abundant antigen-specific Treg cells; **Figure 2**. The advantage of engineered approaches is the reduction of side effects due to nonspecific suppression by Treg cells (103).

Traditional gene transfer approach employed retroviruses, lentiviruses, adenovirus-associated virus (AAV) and transposases which dispense specific and randomized insertion of targeted genes in Treg cells (107, 108). However, randomized insertion by transposases could raise safety concerns (109). Other gene editing or nucleases approaches such as transcription activator-like effector nucleases (TALEN), Zinc finger nucleases (ZFN) and more recently CRISPR-Cas applications advancing Treg therapy and its product (110, 111).

TCR engineered Treg cells are multi protein complex consisting of α- and β-chain which recognize major histocompatibility complex (intra/extracellular antigen) and trigger CD3 dimer complexes (CD3ϵ, CD3γ, and CD3ζ chains) (103). TCR engineered Treg cells provide bystander and antigen-specific suppression by assembling itself in the targeted tissue during autoimmunity (112). Expression of single antigen in a cell is adequate for TCR engineered Treg cell activation but its recognition capacity is limited over a specific population. In the multiple sclerosis mouse model of experimental allergic encephalomyelitis (EAE), myelin basic protein (MBP) activated human Treg cells control the expression of T-effector cells with enhanced Foxp3 expression (113). Further, human TCR transduced for factor VIII (FVIII) also showed dominance over FVIII-specific T-effector cell proliferation and its cytokine production. Moreover, antibody production was also inhibited in antigen-specific manner in FVIII-deficient mice (114). Additionally, transduced Bet v 1-specific Treg cells were more potent than regular tTreg in suppression of allergen-specific T-effector cell proliferation and cytokine production (115). For the future TCR therapy, TCR should be considered from Treg instead of effector T cells.

CAR are the single protein complex consisting of single chain variable fragment which recognize antigen in the outer part, a hinge and inner part with CD3ζ and CD28/4-1BB domain. Elegant work performed by the group of Eshhar licensed the innovation of CAR (116). Initially based on its stimulatory domain, CAR have been alienated into several generations. First generation poses single stimulatory domain (CD3ζ) followed by second generation which display co-stimulatory domain (CD3ζ and CD28), third generation (CD28, CD27, 4-11BB, OX40, ICOS) and fourth generation encompasses with ScFv-CD3ζ, CD28, and 4-1BB (117).

CAR offer direct and broad-spectrum recognition unlike TCR (MHC restricted) and have limited IL-2 dependency but require a high number of targeted tissue antigens (100–10,000 antigens/cell) (118, 119). They can spot entire proteins, major histocompatibility complexes and extracellular antigens (103). CAR engineered Treg specific to HLA-A2 prevent HLA A2^+^ skin rejection and graft versus host disease in mouse models (120, 121). However, CAR recognized extracellular antigens, therefore new tools which combined TCR, and CAR have been developed (122). For instance, the modular chimeric antigen receptor approach unveils suppression functions of co-stimulatory molecules (CD137 and CD28) in Treg and limits the T-effector cells (123). In another study, improved pancreatic islet allograft in mAb-directed CAR expressing Treg cells towards MHC-1 peptides and circumvents skin rejection was monitored (124).

CAR T cells have been utilized for B-cells autoantibodies (IgG) in Pemphigus vulgaris (PV) where CAAR expressing Dsg3 (keratinocyte adhesion protein) specifically targeted autoreactive B cells without influencing other cell types and protected through CD137 signaling cascade (125, 126). Since IgE is a major contributor in the pathogenesis of allergic disease, therefore targeting IgE producing memory B-cells possess great potential for allergic and autoimmune diseases. In this regard, CAR-T-IgE containing α-chain of IgE receptor and FCϵRI displayed specificity towards mouse IgE-B-cell and could be adapted to the clinical settings (127, 128). In asthma, autoantibodies for cell-junction, endothelial and epithelial cells have already delineated which could also be targeted in future (129, 130). In asthma mouse model, mobilization of CAR-Treg cells towards lungs and tracheobronchial lymph node diminished airway hyperreactivity, reduced mucus secreting cells, eosinophilic activity, Th2 cytokines and allergen-specific IgE to control asthma (131). Recently, CAR approach was employed in an allergic model where ovalbumin (OVA) antigen was targeted for mast cells (IgE-sensitized) and B-cells, termed as B-cell Antibody Receptor (BAR). Post OVA challenged for anaphylactic reaction, OVA-BAR expressing Treg cells shielded mice from hypothermia (132). However, human clinical studies using CAR-Treg cells in allergic inflammation are missing so far.

Taken together, Treg therapies and its applications are entering into an exciting era but still confronted with some limitations such as engraftment, safety concerns and long term stability without acquiring plasticity. However, adverse effect or cytokine storm from engineered CAR-Treg cells could be controlled with CRISPR-Cas9 suicidal system containing pro-apoptotic suicidal gene (128, 133). Stability and plasticity, post CAR-Treg cells transplantation could likely be resolved with CRISPR applications either by inserting (IL-10, TGF-β) or removal (IL-17, IL-4, IFN-γ, IL-6) of desired genes (134–136). Further, engraftment efficacy to the targeted tissue is potentially enhanced by expressing desired chemokine receptor in CAR-Treg cells in accordance with overexpressed ligand in the targeted tissue as demonstrated in **Figure 2** (103, 137). Most of the promising preclinical data demanded further optimization and characterization of genetically modified Treg therapy with higher efficacy targeting asthma and allergic diseases to offer alternative curative options at least for patients with severe or chronic respiratory diseases in future.

## Author Contributions 

All authors listed have made a substantial, direct, and intellectual contribution to the work, and approved it for publication.

## Funding

This work was supported by Deutsche Forschungsgemeinschaft (DFG), grants BE 4504/3-3.

## Conflict of Interest

The authors declare that the research was conducted in the absence of any commercial or financial relationships that could be construed as a potential conflict of interest.

## Publisher’s Note

All claims expressed in this article are solely those of the authors and do not necessarily represent those of their affiliated organizations, or those of the publisher, the editors and the reviewers. Any product that may be evaluated in this article, or claim that may be made by its manufacturer, is not guaranteed or endorsed by the publisher.
